# Fetal dystocia in guinea pigs: A case report

**DOI:** 10.29374/2527-2179.bjvm002024

**Published:** 2024-06-27

**Authors:** Julia Penna de Andrade, Sabrina de Morais Miranda, Camilla Faria Soares, Thaís Larissa Lourenço Castanheira, Bruno Ferrante, Marcelo Pires Nogueira de Carvalho

**Affiliations:** 1 Veterinarian, Resident. Programa de Residência em Saúde Pública com Ênfase em Interface Saúde Humana e Silvestres - Escola de Veterinária, Universidade Federal de Minas Gerais (UFMG). Pampulha, Belo Horizonte, MG, Brazil.; 2 Undergraduate in Veterinary Medicine, UFMG. Pampulha, Belo Horizonte, MG, Brazil.; 3 Veterinarian, MSc., Programa de Pós-Graduação em Ciência Animal, Escola de Veterinária, UFMG. Pampulha, Belo Horizonte, MG, Brazil.; 4 Veterinarian, DSc. Departamento de Clínica e Cirurgia Veterinária (DCCV), Instituto Federal do Norte de Minas Gerais. Salinas, MG, Brazil.; 5 Veterinarian, DSc. DCCV, UFMG. Pampulha, Belo Horizonte, MG, Brazil.

**Keywords:** fetal size, obstetrics, rodent, tamanho fetal, obstetrícia, roedor

## Abstract

Dystocia is a common complication in guinea pig pregnancies, presenting significant challenges in clinical management. The present case report describes the presentation, diagnosis, and surgical intervention in an 8 months old female guinea pig with dystocia. The subject is a primiparous guinea pig originating from a commercial breeder, exhibited prolonged labor with two pups, one of which was stillborn. Physical examination revealed a distended abdomen, lack of uterine contractions, signs of distress, and vulvar discharge. Radiographic and ultrasound tests confirmed obstruction due to large fetal size and mineralization of the pubic symphysis. Surgical intervention proceeded with a ventral midline approach, ovariohysterectomy and removal of three fetuses. The guinea pig recovered well from the procedure, being discharged with postoperative care, and the use of anti-inflammatory, analgesics, prokinetics, antibiotics as well as scopolamine. The objective of the present work is to discuss and emphasize the importance of veterinary intervention, diagnostic evaluation and therapeutics for the multifactorial nature of dystocia management. Despite the surgical treatment, the prognosis for both dam and offspring remains guarded, highlighting the need for early detection and intervention to optimize outcomes in guinea pig dystocia cases.

## Resumo

A distocia é uma complicação comum em gestações de porquinhas-da-índia, apresentando desafios significativos no manejo clínico. Este relato de caso descreve a apresentação, diagnóstico e intervenção cirúrgica em uma porca-da-índia fêmea de oito meses que estava experimentando distocia. A porquinha-da-índia, primípara e proveniente de um criador comercial, apresentou trabalho de parto prolongado com dois filhotes, um dos quais nasceu natimorto. O exame físico revelou abdome distendido, falta de contrações uterinas, sinais de angústia e secreção vulvar. Exames radiográficos e de ultrassom confirmaram obstrução devido ao tamanho fetal grande e mineralização da sínfise púbica. A intervenção cirúrgica prosseguiu com uma abordagem ventral na linha média, ovariohisterectomia e remoção de três fetos. A paciente se recuperou bem do procedimento e recebeu alta com cuidados pós-operatórios, incluindo o uso de anti-inflamatórios, analgésicos, pró-cinéticos, antibióticos e escopolamina. O objetivo deste trabalho é discutir e destacar a importância da intervenção veterinária, avaliação diagnóstica e terapêutica na natureza multifatorial do manejo da distocia. Apesar do tratamento cirúrgico, o prognóstico tanto para a mãe quanto para a prole permanece reservado, ressaltando a necessidade de detecção e intervenção precoces para otimizar os resultados em casos de distocia em porquinhas-da-índia.

## Introduction

The guinea pig (*Cavia porcellus*) belonging to the order Rodentia, is widely recognized as a medium-sized rodent and is popular as a non-conventional pet (Smith, 2012). Gestation in this species typically ranges between 68-72 days, with labor lasting approximately 30 minutes, interspersed with breaks of 3-7 minutes for the delivery of subsequent fetuses (Czarnecki & Adamski, 2016). Puberty is reached within 55 to 70 days, with females exhibiting their first estrous cycle by 89 days (Couto, 2006; Lapchik et al., 2017), although both male and females can reach maturity and fertility before 2 months of age (Genzer et al., 2023). Some authors advocate for the initiation of breeding before 6-7 months of age, primarily to mitigate the risk of dystocia, attributed to the stiffening of the pubic symphysis, which interferes with relaxation during birth. Studies have reported varying outcomes based on parental age, with adults showing higher fetal delivery rates, while juveniles present an elevated survival rate (Genzer et al., 2023). Litter size ranges from one to eight fetuses, with an average of 2.75 offsprings (Couto, 2006), each pup weighing between 60-100g (Kaiser et al., 2010). The primary causes of dystocia include pelvic symphysis fusion in unbred females and large fetal size, leading to a poor prognosis due to late detection, often requiring caesarean section. The objective of the case report herein is to review the caesarean section and ovariohysterectomy performed on an 8 –months old guinea pig pregnant with five fetuses, along with the subsequent follow-up treatment.

## Case description

An 8 –months old female guinea pig weighing 880g was scheduled for an appointment due to dystocia. The subject came from a commercial breeder and this was her first litter. The tutor reported a labor duration of 12 hours, during which the guinea pig delivered two pups, one live and one stillborn. The following vital parameters were assessed: pulse rate at 150 beats per minute, respiratory rate at 88 breaths per minute, 39°C temperature, and 10% dehydration, with a glucose level of 108mg/dL. Physical examination revealed a significantly distended abdomen, firm on palpation, with no evident uterine contractions. The tutor had administered calcium and oxytocin, obtained without veterinary prescription and in unknown dosages, prompted by advice from social media. Upon failure of this unguided treatment, the guinea pig was brought in for a veterinary appointment, exhibiting signs of distress, prostration and pain, with a bloody and mucoid vulvar discharge. Given the severity of the situation, surgical intervention was deemed necessary, despite specific rates of surgical necessity in guinea pig dystocia cases not being well-documented when compared to dogs and cats (Gilson, 2003). Imaging tests were requested to assess fetal distress and determine the surgical intervention demand. Blood samples could not be collected prior to surgery because the in-hospital laboratory was already closed.

**Figure 1:**
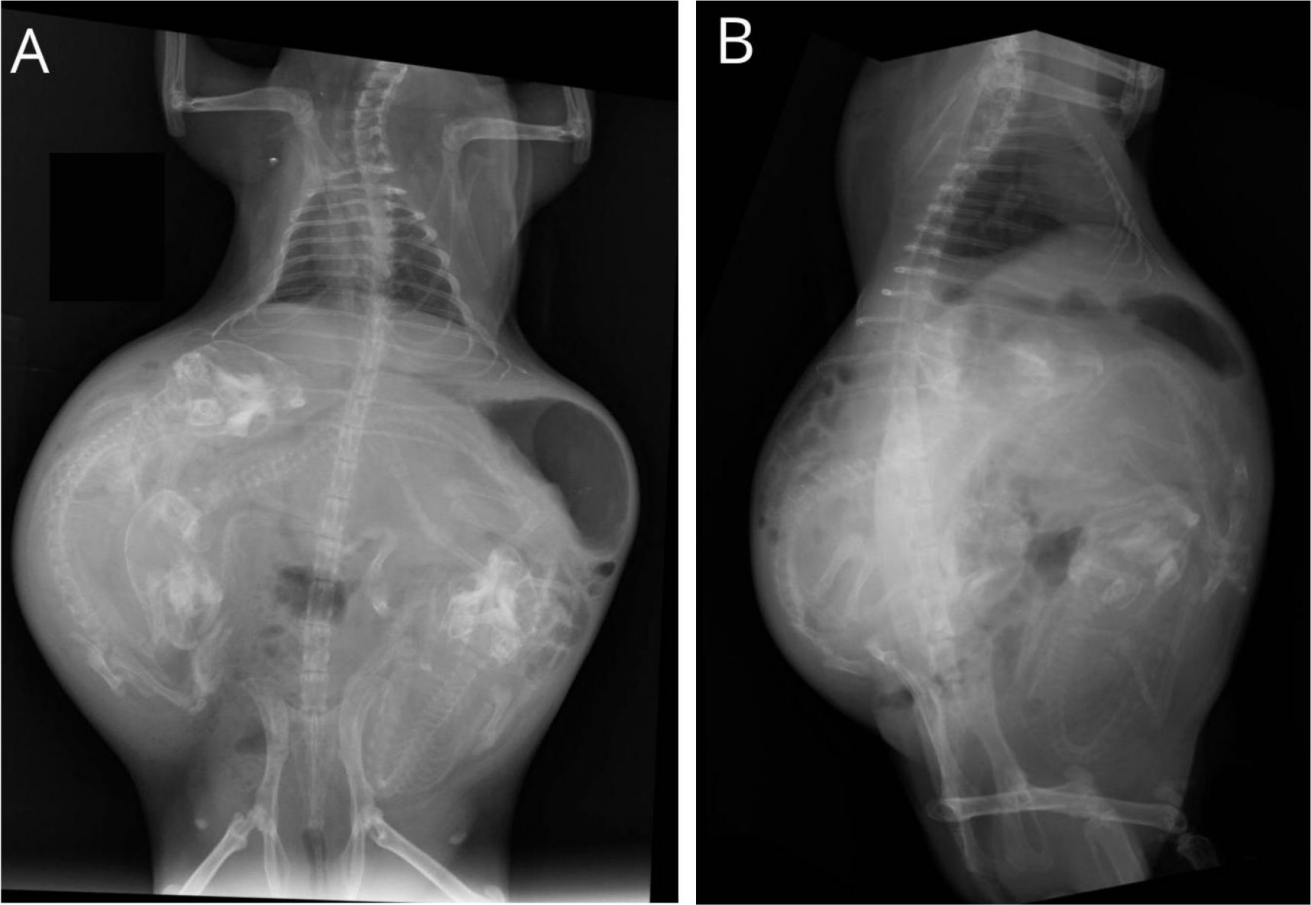
Radiography of *Cavia porcellus* (female, 8 months and 880g) presenting dystocia. (A) Ventro-dorsal projection showing three large, mineralized fetuses at risk of dystocia. (B) Left latero-lateral projection. Source: Diagnostic Imaging Center of the Federal University of Minas Gerais (*Setor de Diagnóstico por Imagem* - UFMG).

Radiographic examination revealed the presence of three fetuses with mineralized skeletons (Figure 1), indicating a physical obstruction likely attributable to fetus size and pubic symphysis mineralization. Ultrasonography further confirmed the lack of fetal viability, evidenced by the absence of heart rate and uterine contractions, consistent with prolonged labor and fetus distress. Given the ineffectiveness of prior clinical treatments and the impracticability of pelvic delivery due to fetal size and labor duration, surgical intervention was deemed necessary. Prior to surgery, the guinea pig was premedicated with enrofloxacin 5% (10mg/kg IM), meloxicam 0.2% (0.5mg/kg IM), dipyrone 500mg/mL (25mg/kg PO), and fluid therapy with ringer (20ml/kg SQ). Anesthesia was induced with diazepam (0.5mg/kg IM), ketamine (5mg/kg IM), morphine (2mg/kg IM), and maintained trans-surgically with inhaled sevoflurane (2%).

The surgical room was meticulously prepared with a backhaus forceps, antisepsis was achieved with degerming chlorhexidine 2%, and alcoholic chlorhexidine solution 0.5%. A ventral midline approach was used for the surgical incision, revealing a thin *linea alba* due to the distension of the muscular wall. Upon the exposure of the uterus, the right uterine horn appeared to be significantly enlarged than the contralateral side, accommodating three fetuses. Remarkably, the left uterine horn showed no evidence of pregnancy, indicating that all five fetuses were situated in the right horn. Due to the heightened risk of muscle laceration, the incision was carefully enlarged to facilitate the exteriorization of the uterus, horns, and removal of the stillborns. Ovariohysterectomy was then performed, involving ligation of the ovarian pedicles, uterine horns and uterus with poliglecaprone 25 3-0 (Gilson, 2003). Subsequent cavity inspection ensured hemostasis, followed by myorrhaphy and dead space reduction using reverdin and a simple continuous suture technique employing poliglecaprone 3-0. Dermorrhaphy was performed with an inverted X pattern with nylon 4-0.

**Figure 2:**
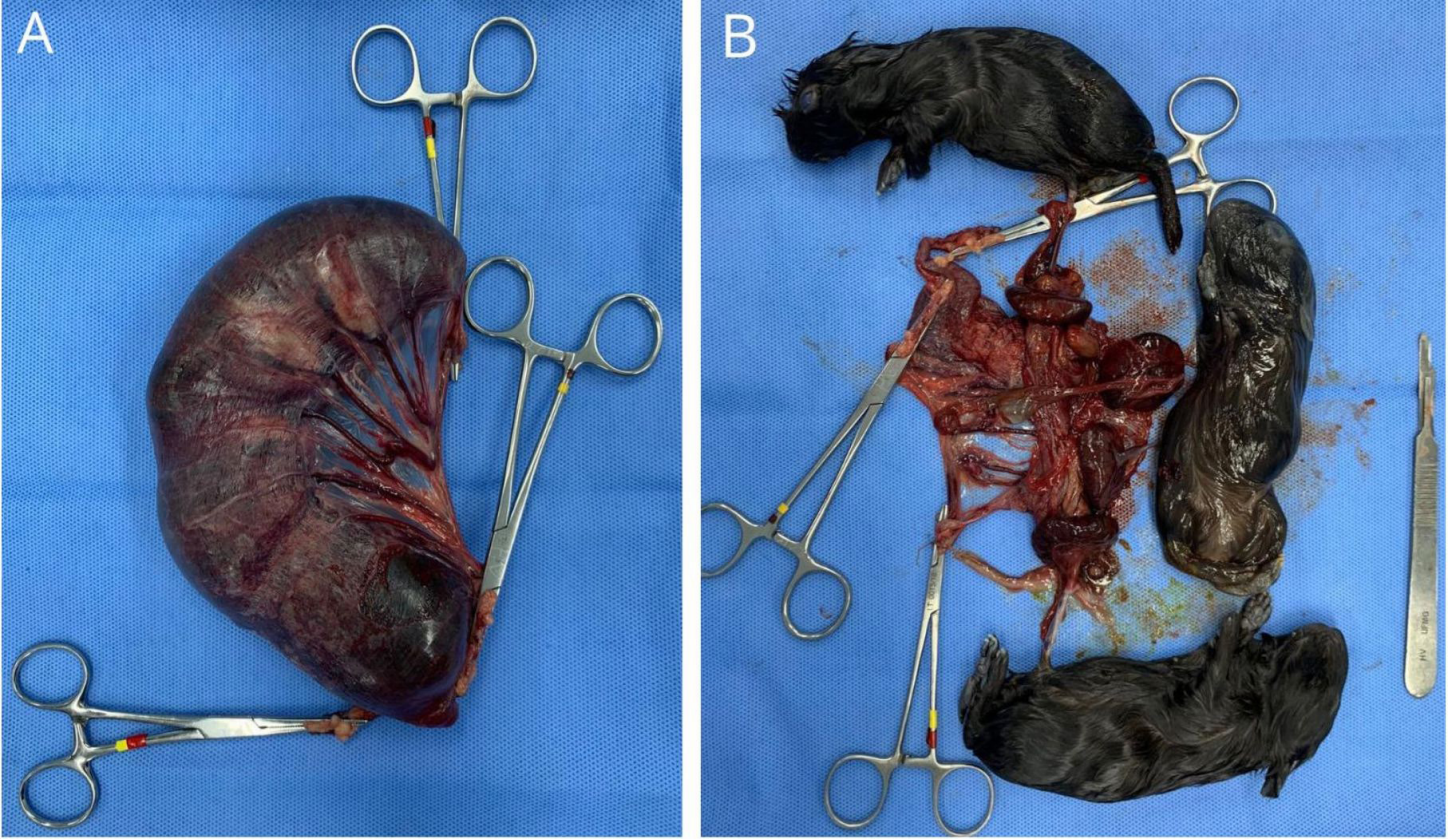
Evaluation of the uterus horn and fetus from a *Cavia porcellus* (female, 8 months and 880g) presenting dystocia. (A) Right uterus horn presenting dilatation, enlarged veins (white arrows) and dark red color with lacerated areas (black arrows). (B) Three stillborn fetuses weighing 350g. Source: Veterinary Hospital of the Federal University of Minas Gerais (*Hospital Veterinário* - UFMG).

The right uterus horn evaluation revealed three fully developed pups, totalling 350g in weight (Figure 2). However, the uterus exhibited severe dilation, presenting a dark red appearance with enlarged veins, evidence of rupture and laceration (Figure 2A). One of the pups displayed signs of maceration, including skin discoloration and peeling (Figure 2B). A surgical dressing was applied using sterile gauze and Micropore tape, and the removal of stitches was scheduled in 10 days. Fortunately, the postoperative period was uneventful, with the guinea pig promptly recovering from anesthesia, resuming normal eating and defecation. The animal was closely monitored for 72 hours before being discharged home. Postoperative therapy included enrofloxacin 5% (10mg/kg/SID/5d IM), meloxicam (0.5mg/kg/SID/3d IM), dipyrone (25mg/kg/TID/3d PO), simethicone (75mg/kg/TID/3d PO), and metoclopramide (0.5mg/kg/BID/3d IM).

## Discussion

The widening of the pubic symphysis is crucial to accommodate the larger dimensions of the fetus during parturition. However, in females older than 8 months, this widening process is significantly impaired due to pubic symphysis mineralization, thereby increasing the likelihood of requiring medical intervention (Hawkins & Bishop, 2012). In the present case report, the female guinea pig was already 8 months old, a compounded factor which contributed to her dystocia.

Dystocia is characterized by the inability to undergo normal labor and can be caused by many contributing factors, including premature fetuses, obesity, uterine inertia or torsion, as well as risks associated with the pups, such as large fetal size and malposition (DeCubellis, 2016; Hawkins & Bishop, 2012). In the case herein, dystocia appears to be attributable to two primary factors. Firstly, the eight months old female was beyond the recommended age for first parturition, which may have contributed to complications. Secondly, the fetuses were notably large for the species, with an average weight of 115g, when compared to the typical range of 60 to 100g for guinea pig fetuses. Remarkably, the combined weight of the five fetuses represented approximately 63% of the mother's body weight (Kaiser et al., 2010). It is possible that these factors increased the risk of dystocia in this case.

The pubic symphysis in guinea pigs does not revert to its fibrocartilaginous state after parturition. Instead, it remains in a fibrous ligamentous state. As a result, the risk of obstructive dystocia is significantly reduced in subsequent deliveries of the sow (Hawkins & Bishop, 2012; Ortega et al., 2003). Eventhough the recommended therapy varies according to the underlying cause, the clinical treatment is mostly associated with oxytocin and calcium gluconate, as well as surgical intervention with caesarean section along with ovariohysterectomy (Lange & Schmidt, 2014).

In the present case report, the tutor took independent measures to address the prolonged parturition interval between the first successful birth and the subsequent stillborn delivery. Administring unknown doses of oxytocin and calcium without veterinary guidance, the tutor attempted to expedite the birthing process. However, these interventions proved to be ineffective, as the primary cause of dystocia was obstructive. Ecbolic agents like oxytocin are intended to induce delivery within 30 minutes of administration, but their efficacy may be compromised by factors such as muscular fatigue and hypocalcemia (Kondert & Mayer, 2017; Paul-Murphy, 2011). Moreover, the inappropriate use of oxytocin in this case posed risks of uterine muscle exhaustion and rupture, as evidenced by the rupture and laceration areas observed in the uterus (Blaes, 2001).

The correct protocol in this case would have involved a prompt clinical assessment of the female upon the manifestation of evident signs of exhaustion and labor incapacity, followed by imaging tests to evaluate fetal viability (Brower, 2006). Notably, while pup sizes typically increase as the number of birth decreases, all the pups in this case exhibited weights between 100-115g, rising the chances of dystocia attributable to fetal size. Surgical intervention may have been warranted in cases of fetal obstruction, as supported by relevant literature (Biddle & MacIntire, 2000; Jutkowitz, 2005).

Even if the appropriate protocol had been followed, the prognosis for both the female and the pups would remain poor. The high oxygen demand of fetuses and their low tolerance for carbon dioxide present a significant risk factor for hypoxemia in prolonged labor (Brower, 2006; Pollock, 2017). In guinea pigs, dystocia is expected when labor duration exceeds 30 minutes and should be confirmed through radiography and ultrasounds. An accurate determination of the main cause is crucial for implementing the proper treatment measures.

Surgical intervention is indicated in cases of mineralization of the pubic symphysis and fetal dystocia with no possibility of natural delivery (Brower, 2006). The surgical protocol should be performed when clinical therapy is not feasible, and sterilization is recommended to prevent further pregnancies (Paul-Murphy, 2011). The surgical treatment implemented herein is in accordance with the literature, involving midline incision or unilateral flank incision, with ligation and dissection of the ovaries, uterine horns and uterine cervix (Rosanska et al., 2016). Antibiotic therapy is indicated in cases of suspected non-sterile procedures and in cases of prolonged labor, due to ascending bacterial infection (Paul-Murphy, 2011). The lack of hematological and biochemical analysis in this case precluded the confirmation of uterine infection. Despite the surgical procedure being successful, the prognosis is unfavorable for animals requiring surgical treatment, as prevention is the most successful approach (Brower, 2006).

## Conclusion

In conclusion, the present case report emphasizes the complexities and challenges associated with dystocia in guinea pigs, particularly in cases involving obstruction due to large fetal size and inadequate pubic symphysis widening. The report herein stresses the importance of a timely veterinary intervention along with a proper diagnostic evaluation, including imaging tests to assess fetal viability and determine the most appropriate therapeutic approach. Furthermore, this case report highlights the risk of an unguided administration of drugs, such as oxytocin and calcium, reinforcing the necessity of veterinary supervision to manage dystocia cases. Despite the surgical intervention in this case, the prognosis for both sow and pups remain guarded, indicating the need for early detection and intervention in dystocia cases to optimize outcomes.
